# Mental and physical health in prison: how co-occurring conditions influence inmate misconduct

**DOI:** 10.1186/s40352-018-0082-5

**Published:** 2019-01-05

**Authors:** Daniel C. Semenza, Jessica M. Grosholz

**Affiliations:** 1 Department of Sociology, Anthropology, & Criminal Justice, Rutgers University, 405-7 Cooper Street, Camden, NJ 08102-1521 USA; 2 College of Liberal Arts and Social Sciences, University of South Florida Sarasota-Manatee, Sarasota, FL USA

**Keywords:** Inmate misconduct, Physical health, Mental health, Co-occurring disorders, General strain theory

## Abstract

**Background:**

Research has shown that inmate misconduct is related to a range of demographic factors and experiences with the criminal justice system. Poor mental and physical health has also been associated with inmate misconduct, although no research has examined the relationship between co-occurring conditions and misconduct in prison populations.

**Methods:**

We rely on data from the 2004 Survey of Inmates in State Correctional Facilities (*N* = 14,499) and use negative binomial regression models to examine the relationship between types of co-occurring mental and physical conditions and misconduct.

**Results:**

The results demonstrate that people in prison dealing with concurrent mental and physical health problems are significantly more likely to engage in prison misconduct than healthy incarcerated individuals. After accounting for physical and co-occurring health conditions, mental conditions are not associated with serious misconduct.

**Conclusions:**

Enhancements in prison healthcare may not only improve the general health of those in prison, but also contribute to a decrease in misconduct. Research that examines the relationship between mental health and deviant behavior in and out of prison should consider the multifaceted elements of a person’s health, including acute and chronic physical ailments.

Due to the deinstitutionalization of mental health hospitals across the United States (U.S.) over the last fifty years, the U.S. prison system has witnessed an increase in the number of those in prison with mental disorders (see Primeau et al., 2013) with research suggesting that there are 10 times more individuals with a mental disorder in prison or jail than housed in mental hospitals (Haney, 2017; Torrey et al., 2014). In addition to this significant rise in mental disorders behind bars, rates of co-occurring disorders are striking.^1^ Past investigations find that of those in jail with severe mental disorders, there is a 72% rate of co-occurring substance abuse (Abram & Teplin, 1991). More recent evidence also suggests that people in prison with a mental disorder are more likely to experience substance abuse than those without (Mumola & Karberg, 2006). Corrections scholars find that people in prison who suffer from co-occurring disorders are more likely to engage in violence and misconduct, as well as become victims of such aggression (Friedmann et al., 2008; Houser et al., 2012; Houser & Welsh, 2014; Wood, 2012, 2014; Wood & Buttaro Jr., 2013). Researchers typically describe an individual as suffering from a co-occurring disorder if they experience both a mental disorder and substance use disorder. However, no research has examined how suffering from a mental disorder combined with a physical condition influences prison behavior despite the fact that those in prison have worse physical health than their non-institutionalized counterparts (see Aday, 2003; Loeb et al., 2008; Williams & Abraldes, 2007).

Given significant health disparities in prison populations and the ongoing effects of deinstitutionalization, many prisons now face inmate health concerns that include co-occurring chronic and acute ailments, physical disabilities, and mental disorders. Using Agnew’s General Strain Theory (GST) as an overarching theoretical framework, we argue that experiencing co-occurring forms of mental and physical conditions in prison creates significant accumulated strain, potentially leading to misconduct. We first review GST and discuss the relationship between cumulative, co-occurring health strain and institutional misconduct. We then discuss our methodology, analysis and results. We conclude with important policy implications for institutional misconduct and correctional healthcare.

## Background

### General strain theory and health

According to General Strain Theory (GST), individuals experience three major types of strain: the failure to achieve positively valued goals, the removal of positively valued stimuli, and the presence of negative stimuli (Agnew, 1992). These stressors then lead to negative emotions like anger, depression, and frustration (Agnew, 1992). Without the appropriate, legitimate coping mechanisms to alleviate these feelings, individuals may turn to illegitimate or criminal avenues (Agnew, 1992, 2001, 2006). While Agnew (1992) developed GST through a social psychological lens to expand upon Merton’s (1938) original strain theory and explain juvenile delinquency, the theory has been employed to examine a multitude of criminal and delinquent behaviors. Throughout the years, the relationship between strain and crime has found continued empirical support, suggesting that as strain increases, one’s likelihood of engaging in crime or delinquency also increases (e.g., Baron, 2004, 2006; Broidy, 2001; Cullen et al., 2008; Ford & Schroeder, 2009; Manasse & Ganem, 2009; Schroeder & Ford, 2012; Stogner & Gibson, 2010, 2011; Zavala & Spohn, 2013).

One major type of strain that encompasses all three of Agnew’s domains is poor health. When an individual has poor health (mental or physical), he or she may not be able to achieve desired goals. This may include the inability to achieve financial security, stability in relationships, or personal independence due to pervasive or disruptive health issues. Poor health may also lead to the loss of positively valued stimuli by making daily functioning more difficult or presenting additional health-related challenges to which the individual is unaccustomed. Last, health problems are inherently noxious stimuli that may create significant strain in a person’s life simply by causing the person discomfort, pain, or anguish.

### Mental and physical health strain

The majority of the current research on health strain and inmate misconduct focuses on the relationship between mental disorder and criminal involvement. This direction of scholarship makes sense considering the high prevalence of mental disorders in the U.S. criminal justice system (see Skeem et al., 2011). Studies find that of the factors found to influence inmate behavior, mental disorder is one of the most consistent and significant predictors of institutional misconduct (Adams, 1986; Carr et al., 2013; Felson et al., 2012; Houser et al., 2012; Matejkowski, 2017; McCorkle, 1995; Steiner & Wooldredge, 2009; Steiner et al., 2014; Stewart & Wilton, 2014; Wood, 2012; Wood and Buttaro Jr., 2013).

Like mental disorders, those in prison also have higher rates of physical health conditions than the public (Bronson et al., 2015; Nowotny et al., 2016; Weinbaum et al., 2005). While little is known about the influence of poor physical health on prison behavior, scholars have examined the bidirectional relationship between physical health strain and delinquency outside of confinement. One area of research, in line with the causality indicated by GST, has found that poor physical health influences criminal involvement, the onset of offending, and subsequent crime escalation (Ford, 2014; Schroeder et al., 2011; Stogner & Gibson, 2011). On the other hand, additional studies have found that criminal involvement increases the likelihood of poor health (Piquero et al., 2007; Piquero et al., 2011). For instance, Piquero et al. (2007) observed that life-course persistent offenders had poorer chronic and mental health later in life than adolescence-limited offenders and non-offenders.

Recent studies have indicated that both acute and chronic physical ailments may influence deviant and criminal behavior, although mixed results suggest that further research is needed to understand the unique influences of different forms of physical conditions. Much of this research has leveraged GST to frame the relationship between physical health problems and offending, suggesting that people may not have the necessary coping resources to deal with newly acquired physical ailments and accompanying negative emotions like anger, stress, and frustration (Stogner & Gibson, 2010). Health problems may present additional noxious stimuli such as disruptions to routines, financial stress, and relationship barriers (Stogner et al., 2014). Poor physical health has therefore been shown to be a strain in and of itself as well as a potential cause of additional strains that can result in deviant behavior.

Recent research highlights the compounding effect of health strain on additional stressors and subsequent delinquency. Kort-Butler (2017) finds that health-related strain is positively associated with delinquency and marijuana use. She also observes that those youth who experience health strain suffer stressors in secondary contexts (e.g., trouble with schoolwork, teachers, etc.), which put them at a greater risk of delinquency and substance use. In line with this argument, then, experiencing poor physical health in prison, an already strained environment, may put physically ill individuals at an increased risk of institutional misconduct.

Researchers have also recently examined the role that physical health problems play in institutional misconduct (Grosholz & Semenza, 2018). Using a framework proposed by Van Gelder (2013), Grosholz and Semenza (2018) suggest that individuals in prison dealing with chronic conditions experience a “cold affect,” which is associated with increased self-control and a better ability to weigh costs and benefits. While these individuals might be angry and frustrated with the state of their health, they may have become accustomed to being ill and have the appropriate coping mechanisms in place to handle the associated strain. Additionally, those in prison with chronic physical conditions may not have the physical ability to engage in misconduct or they may have simply “aged out” of misconduct. On the other hand, those suffering from acute physical conditions are more likely to experience a “hot affect,” which leads to more impulsive behavior and increasingly irrational decisions (Van Gelder, 2013). In line with Van Gelder (2013), they find that people in prison suffering from acute physical conditions – whose symptoms are short-lived – are significantly more likely to engage in misconduct than those who are healthy. Likewise, those experiencing chronic ailments or long-term disabilities, which result in more persistent, long-lasting symptoms, are significantly less likely to engage in misconduct (Grosholz & Semenza, 2018). Thus, poor physical health appears to be a strain associated with misconduct if it is acute in nature rather than chronic. Little remains known about how health problems may combine to create additional strains that may result in misconduct.

### Co-occurring health conditions and institutional misconduct

One can argue that experiencing more than one strain at a time will increase the likelihood of engaging in crime or misconduct. In line with Agnew’s (2006) assertion that strains high in magnitude are more likely to influence crime, Botchkovar and Broidy (2010) highlight the impact of strain accumulation, or the clustering of strains, on criminal involvement (Botchkovar & Broidy, 2010). In particular, they examine the relationship between strain, negative emotions, and illegal coping, concluding that “when exposure to strain is repetitive and routine, accumulation or clustering of negative events and conditions may boost the crime-generating potency of other, less criminogenic strains” (Botchkovar & Broidy, 2010, p.851). Since poor health is likely a significant strain that can lead directly or indirectly to negative emotions, the experience of multiple conditions may be a form of strain accumulation that is particularly high in magnitude and influential for misconduct. Given that a person’s own health is of central importance to their daily functioning, general comfort, and overall well-being, the experience of multiple health problems may be particularly problematic. This may especially be the case if an individual is dealing with both a mental and physical condition, or dealing with acute physical symptoms on top of a chronic condition in a highly deprived, strained environment like prison. In the same way that an individual may experience different forms of strain across various domains in their lives (e.g., work, school, family), they may also have to cope with multiple strains across areas of their own personal health.

As the number of individuals behind bars suffering from co-occurring disorders has increased (Nowotny et al., 2016), corrections scholars have begun to examine the effect of dual diagnoses on behavior. Studies reveal that those suffering with co-occurring mental disorders and substance abuse – a form of accumulated strain in prison – are significantly more likely to engage in misconduct, be victims of violence, and receive harsher disciplinary sanctions (Friedmann et al., 2008; Houser & Belenko, 2015; Houser et al., 2012; Houser & Welsh, 2014; Wood, 2012, 2014; Wood & Buttaro Jr., 2013). Despite documented research, the field traditionally defines co-occurring disorders as a mental disorder combined with substance use disorder. However, a substantial body of literature indicates those with a mental disorder are more likely to have a comorbid physical condition and suffer from greater physical symptoms (Hert et al., 2011; Osborn, 2001; Thornicroft, 2011). Those with a mental health disorder suffer from higher rates of physical health conditions including cardiovascular diseases, diabetes, chronic pain, respiratory diseases, gastrointestinal illnesses, and cancer (Hert et al., 2011; Sareen et al., 2007).

While psychiatric disorders are associated with greater physical problems, the two forms of conditions are often inextricably linked, leading to a clustering of health strains. As a result, they should be examined together as they relate to prison behavior. No research to date has accounted for co-occurring physical conditions when assessing the link between mental disorder and misconduct. Given the lack of research, the current study examines the relationship between co-occurring mental and physical health conditions and inmate misconduct. Our research question therefore asks, “How does experiencing a combination of multiple co-occurring health conditions – both mental and physical – affect the risk of inmate misconduct?”

## Data and Methods

### Study design and sample

We use data from the 2004 *Survey of Inmates in State Correctional Facilities* (SISCF). The data include information on a nationally representative sample of individuals in state prisons in the United States and is the most up-to-date survey on this population publically available to researchers. Researchers interviewed a total of 14,499 individuals between October 2003 and May 2004 and used a two-stage, stratified sampling procedure.^2^ Participation in the survey was fully voluntary and interviews covered a wide range of topics including current offense and sentencing information, criminal history, health, and demographic characteristics.^3^ Since these data are cross-sectional in nature, they do not enable a full test of GST using measures of negative emotions like anger or frustration as mediators between poor health and misconduct. Rather, GST is used as a guiding framework for this initial assessment of the relationship between co-occurring conditions and prison misconduct.

### Key measures

#### Inmate misconduct

See Table 1 for information on all individual measures and indices used in this analysis. The main dependent variable is inmate misconduct. Survey researchers asked participants if they had ever been written up or found guilty of the following ten offenses: weapon possession, possession of stolen property, found in possession of an unauthorized object or substance, escape or attempted escape, being out of place, verbal assault of a staff member, verbal assault of another inmate, disobeying orders, drug violation, or alcohol violation. We coded each form of misconduct “1” if the individual indicated that they had been written up or found guilty of that violation and “0” if they did not.

**Table 1:** Unweighted Observed Characteristics of Full Analytic Sample, State Inmates, 2004. (Pre-imputation; *N* = 12,272)

Variables and Items	*M*	*(SD)*	Obs. R
Dependent Variables			
Prison misconduct types (all ref.: no)			
Alcohol violation	0.02	*0.14*	0–1
Disobey	0.25	*0.43*	0–1
Drug violation	0.05	*0.23*	0–1
Escape attempt	0.01	*0.09*	0–1
Out of place	0.15	*0.36*	0–1
Stolen property	0.01	*0.11*	0–1
Unauthorized item	0.14	*0.34*	0–1
Verbal assault of a guard	0.08	*0.27*	0–1
Verbal assault of an inmate	0.05	*0.23*	0–1
Possession of a weapon	0.03	*0.17*	0–1
Non-serious prison misconduct - variety scale, *ct*	0.67	*0.42*	0–5
Serious prison misconduct - variety scale, *ct*	0.13	*1.04*	0–5
Main Independent Variables			
Acute health conditions - variety scale, *ct*	1.43	*1.06*	0–4
Chronic health conditions - variety scale, *ct*	0.67	*1.04*	0–9
Mental health conditions - variety scale, *ct*	0.61	*1.18*	0–7
Categories of co-occurring health conditions			
Chronic + mental	0.02	*–*	*–*
Chronic + acute	0.20	*–*	*–*
Acute + mental	0.10	*–*	*–*
All three conditions	0.13	*–*	*–*
Covariates			
Drug dependence (ref: no)	0.38	*0.49*	0–1
Work assignment (ref: no)	0.62	*0.49*	0–1
Job program (ref: no)	0.28	*0.45*	0–1
Number of prior incarcerations	1.87	*4.58*	0–161
Total months served	55.62	*63.23*	0–523
Sentence length (months*)*	125.42	*180.68*	0–4400
Offense type			
Violent	0.46	*–*	*–*
Property	0.20	*–*	*–*
Drug	0.23	*–*	*–*
Public order	0.11	*–*	*–*
Race			
White	0.37	*–*	*–*
Black or African American	0.40	*–*	*–*
Hispanic	0.17	*–*	*–*
Other/multiple races	0.05	*–*	*–*
Female (ref: male)	0.20	*–*	*–*
Age	35.33	*10.43*	16–84
Education	10.87	*2.32*	0–18

*ABBREVIATIONS: ct* = count. *M* = mean. (*SD*) = standard deviation. *Obs. R* = observed range

*ref* = reference group

*NOTE:* Results shown for individual scale items first, followed by full scale results. Mean and standard deviations are reported for continuous variables only

We used these ten items to create two separate scales to measure (1) serious and (2) non-serious misconduct. The serious misconduct scale includes: drug violation, alcohol violation, weapon possession, possession of stolen property, and escape or attempted escape (α = 0.61). The non-serious misconduct scale includes: possession of an unauthorized object, verbal assault of a staff member, verbal assault of another inmate, being out of place, and disobeying orders (α = 0.60). Mean engagement for the majority of misconduct types is unsurprisingly low, though disobeying orders (25%), being out of place (15%), and having an unauthorized item (14%) have the highest rates of sanction. Non-serious misconduct is more prevalent among the sample population (*M* = 0.67; *SD* = 0.42), compared to serious misconduct (*M* = 0.13; *SD* = 1.04). Recent research suggests that separating items into serious and non-serious misconduct is appropriate given that there may be very different risk factors and consequences for particular subgroups of misconduct behaviors (Gendreau et al., 1997; Grosholz & Semenza, 2018; Rocheleau, 2013). Although we acknowledge that some of these misconduct types may be more or less serious depending on their specific context (e.g., the type of unauthorized object possessed), these two categories indicated the greatest inter-item reliability for all combinations of misconduct assessed.^4^

We use variety measures of misconduct (indices of different types of misconduct violations, rather than the sum of the number of times violations are committed) because they demonstrate greater stability over time and improved internal consistency when compared to frequency measures (Bendixen et al., 2003; Grosholz & Semenza, 2018; Sweeten, 2012). Although both measurement types have been verified in criminological research, variety measures are better suited for assessing the prevalence of specific types of misconduct as well as the range of offenses that occur (Steiner et al., 2014; Wolff et al., 2006). Frequency measures produced lower alpha coefficients for both serious misconduct (0.48) and non-serious misconduct (0.57) when compared to analogous variety measures.

#### Mental and physical health conditions

We created scales for three types of health conditions: mental, acute physical, and chronic physical. Each of these captures a variety of conditions within each category. For mental health, researchers asked survey respondents whether they had ever been diagnosed with the following disorders: anxiety, depression, manic depression, bipolar disorder, or mania, a personality disorder, schizophrenia or another psychotic disorder, post-traumatic stress disorder, or any other mental disorder. Each was coded “1” if the individual had been diagnosed. We then added all seven mental health items together.

Researchers asked individuals whether they had any of the following problems since admission: had an illness including a cold, virus, or the flu, incurred an injury due to an accident, dental problems, or had surgery. We consider each of these items to be of an acute nature, rather than a chronic problem. That is, they are not serious ongoing conditions and likely affect the inmate only temporarily, compared to the chronic conditions measured. We coded responses as “1” if the individual indicated that they had dealt with the issue since admission. We then created a measure of acute conditions by adding all four items together.

Finally, researchers asked participants whether they are currently dealing with problems related to the following chronic physical health issues: arthritis, asthma, cancer, cirrhosis, diabetes, heart disease, hepatitis, high blood pressure, kidney problems, paralysis, sexually transmitted disease, or stroke. We coded indication of each chronic condition as “1.” We then created a scale by adding the twelve items together. We include these three variety scales in all models to examine the magnitude of physical conditions experienced by respondents – the greater the number of conditions, the sicker that person is within each category.

Acute health problems are the most prevalent type of condition (*M* = 1.43), followed by chronic (*M* = 0.67) and mental conditions (*M* = 0.61). It is possible that particular health conditions relate to misconduct in diverse ways. For instance, suffering from schizophrenia versus having an anxiety disorder may produce very different outcomes related to institutional misconduct. The same may be the case for different physical conditions, such as having a temporary illness versus dental problems. These individualized relationships have been explored in prior research on the influence of health conditions for both delinquency (Stogner & Gibson, 2010, 2011) and misconduct (Grosholz & Semenza, 2018). Although particular health conditions may relate to inmate misconduct in different ways, we are specifically focused in this study on the unique influence of comorbidity across health condition types for misconduct. As a result, we include these indices of health conditions in all models alongside the measure of co-occurring health conditions.

#### Co-occurring health conditions

To measure the experience of co-occurring health conditions, we recoded each of the three health scales into a binary measure indicating whether the respondent had any of the conditions within that type (acute, physical, or mental; 1 = yes; 0 = no). Thus, it is possible that those in prison may have multiple diagnosed conditions within these mutually exclusive types. We combined the binary measures of mental, acute physical, or chronic physical health conditions to measure the unique combinations of co-occurring conditions experience using the “group” function in Stata. We created mutually exclusive categories of health conditions to signify co-occurring health status: chronic + mental condition, chronic + acute condition, acute + mental condition, and all three categories. Each of these groups is compared to those without any condition as the reference category to assess the effects of dealing with co-occurring types of conditions in prison. The most common category of co-occurring conditions is an acute problem and a chronic condition (20%), followed by an acute and a mental condition (10%), and suffering with a chronic ailment and mental condition (2%). About 13% of the sample indicates experiencing all three types of co-occurring health conditions.^5^

### Covariates

#### Drug dependence, criminal history and current sentencing

Prior research has identified a wide range of diverse prison misconduct correlates (Gendreau et al., 1997; Steiner et al., 2014). We included a binary measure of whether the respondent currently had any drug dependence, including alcohol, in all models. Although drug dependence has been conceptualized in prior research as a significant component within co-occurring disorders (Houser et al., 2012), we used it here as a control to ensure no overlap with our measure of co-occurring health conditions. We included a categorical measure of the individual’s current offense. The available categories included: violent offense (reference), property offense, drug offense, and public order offense. We included the number of prior incarcerations, current sentence length, and the number of months served on the current sentence as continuous variables in all models. We included two binary items to measure whether the respondent currently had a work assignment or participated in a job program.

#### Demographic characteristics

We controlled for certain demographic differences in all models. The respondent’s educational attainment was measured using the highest grade level attended prior to incarceration. Educational attainment ranged from less than kindergarten through graduate education at yearly intervals. Age was measured as a continuous variable. Gender was measured as a dichotomous variable (0 = male; 1 = female). The survey measured self-reported race using the following categories: White, Black or African American, Hispanic, American Indian or Native Alaskan, Asian, Hawaiian or Pacific Islander, or any other race. We created a categorical variable where White was the reference category (coded “0”), Black or African American was coded “1,” Hispanic was coded “2,” and the rest of the categories were combined to create an “Other” designation given low counts in each of the individual categories (< 5% combined; coded “3”).

### Analytic strategy

We first conducted multiple imputation using chained equations (MICE) for all variables to decrease the risk of bias from excluded cases (Little et al., 2013; Schlomer et al., 2010). MICE fills in missing values for multiple variables using a sequence of univariate imputation methods. This method preserves the distribution of all variables in the analysis via fully conditional specifications (StataCorp, 2015; van Buuren, 2007; van Buuren et al., 1999). After accounting for missing data on all variables in our models, approximately 15% of the data was missing. Research suggests that multiple imputation is appropriate when missing data results in a reduction of more than 10% of the data (Cheema, 2014; Langkamp et al., 2010). We generated 20 imputations via the “mi impute chained” command in Stata. This resulted in a full analytic sample of 14,499 respondents.

Following imputation, we conducted all analyses using Stata 14.2. We used negative binomial regression due to over dispersion^6^ in the dependent variable and the frequency of “no misconduct” responses in the sample (60%). We used sample weights included with the data to account for the multi-stage sampling design of the study. We first regressed the variety measure of general misconduct on all variables. We then regressed measures of non-serious misconduct and serious misconduct on all variables. We examined our research question using the co-occurring health conditions measure across all three types of misconduct. All results are reported using incidence rate ratios (IRR’s).

The co-occurring health conditions measure is included alongside the three types of health condition scales to examine the unique influence of co-occurring health problems after accounting for the variety of conditions experienced in each category. This approach enables us to parse out whether combinations of conditions create greater risk for misconduct. To ensure no problems of multicollinearity, we conducted variance inflation factor (VIF) tests for all independent measures. Although there is no universally accepted cut-off point, recent estimates suggest that VIF scores below 5 indicate minimal multicollinearity (Kutner et al., 2004; O’Brien, 2007; Vatcheva et al., 2016). The highest VIF score calculated for all independent variables was 2.53 for the co-occurring health conditions measure, suggesting confidence in limited multicollinearity for the models used here.

## Results

Table 2 provides a correlation matrix for the dependent measures and all key health-related independent variables. Though the measure of co-occurring health conditions is significantly correlated with each of the three specific health condition indices (acute, chronic, mental), it is a distinct measure of combinations that is moderately correlated with the particular condition type measures (0.65 for acute conditions is the largest correlation). Given low VIF scores, as noted above, and a bivariate correlation cutoff point of .8, we encountered no issues of multicollinearity during multivariate analysis (Berry & Feldman, 1985).

**Table 2:** Unweighted Correlation Matrix for Key Variables, State Inmates, 2004. (Pre-imputation; N = 12,272)

Measure		1	2	3	4	5	6
1	Non-serious misconduct	–					
2	Serious misconduct	0.36***	–				
3	Mental health conditions	0.09***	0.03***	–			
4	Chronic health conditions	0.02***	0.01	0.25***	–		
5	Acute health conditions	0.29***	0.21***	0.08***	0.18***	–	
6	Co-occurring health conditions	0.19***	0.11***	0.44***	0.37***	0.65***	–

*p < .05; **p < .01; ***p < .001 (two-tailed)

Table 3 provides the weighted multivariate results for non-serious misconduct and serious misconduct. Acute physical conditions are associated with an increase in non-serious misconduct by about 25% and serious misconduct by 30%. Having a chronic physical condition is associated with a decrease in misconduct across both models as well. This is most pronounced for serious misconduct, where having a chronic condition is associated with a reduced likelihood of misconduct by about 13%.

**Table 3:** Weighted Negative Binomial Regressions of Inmate Misconduct in State Prisons (Imputed; N = 14,499)

	Inmate Misconduct			
Model	*Non-serious*		*Serious*	
Parameter	IRR	(SE)	IRR	(SE)
Acute physical health conditions, ct.	1.25***	(0.02)	1.30***	(0.05)
Chronic physical health conditions, ct.	0.91***	(0.02)	0.87***	(0.04)
Mental health conditions, ct.	1.05**	(0.02)	1.07	(0.04)
Co-occurring health conditions (ref = none)				
Chronic + mental	1.48**	(0.20)	1.37	(0.46)
Chronic + acute	1.22**	(0.10)	1.47*	(0.26)
Mental + acute	1.44***	(0.11)	1.40	(0.26)
All three	1.53***	(0.14)	1.50*	(0.32)
Drug dependence (ref = no)	1.23***	(0.04)	1.68***	(0.10)
Work assignment (ref = no)	0.89***	(0.03)	0.71***	(0.04)
Job program (ref = no)	1.38***	(0.04)	1.48***	(0.09)
Number of prior incarcerations	1.00	(0.00)	1.01	(0.00)
Months served	1.00***	(0.00)	1.01***	(0.00)
Sentence length	1.00***	(0.00)	1.00*	(0.00)
Offense type (ref = violent offense)				
Property	0.87***	(0.03)	0.73***	(0.06)
Drug	0.70***	(0.03)	0.55***	(0.05)
Public order	0.76***	(0.04)	0.76*	(0.10)
Gender (ref = male)	1.03	(0.04)	0.55***	(0.05)
Racial group (ref = white)				
Black	1.25*	(0.05)	1.11	(0.02)
Hispanic	0.93	(0.04)	0.99	(0.10)
Other	1.09	(0.07)	1.04	(0.13)
Age	1.00	(0.00)	1.00	(0.00)
Education	0.96***	(0.01)	0.95***	(0.01)
Constant	0.40***	(0.05)	0.06***	(0.01)
Alpha	0.544		0.676	

*NOTE:* Standard errors are in parentheses next to parameter estimates

*ABBREVIATIONS: IRR* = incidence rate ratio, *ref*. = reference category in binary variable (=0);

*ct*. = count, *SE* = standard error

*p < .05; **p < .01; ***p < .001 (two-tailed)

All combinations of co-occurring health conditions are associated with non-serious misconduct. Dealing with both a mental and chronic physical condition together is associated with about a 48% increase in the likelihood of non-serious misconduct (*p* < .05), compared to those who are healthy. This is notable, given that having a mental condition alone has a rather small association with non-serious misconduct and having a chronic condition alone is associated with a decrease in non-serious misconduct. The results suggest that, in combination with one another, these conditions may create a compounded strain for the person in prison that leads to minor misconduct.

Although co-occurring conditions are more consistently associated with increases in non-serious misconduct, we find certain relationships for serious misconduct. Dealing with a chronic condition and an acute health condition is associated with about a 20% increase in the likelihood of non-serious misconduct (*p* < .001) and about a 50% increase in the likelihood of serious misconduct (*p* < .05). Suffering with a mental and an acute condition is associated with a 44% increased likelihood of non-serious misconduct, though we find no significant relationship with serious misconduct. Finally, experiencing all three types of health conditions is associated with a roughly 50% increase in the likelihood of misconduct for both non-serious and serious misconduct.

## Discussion

Our results suggest support for a relationship between compounded health strain and institutional misconduct. The present findings indicate that, in addition to the strain of dealing with one type of condition, those in prison may experience a form of compounded strain in which co-occurring conditions increase the likelihood of misconduct. Cumulative health strain is likely worsened by being in prison, where the individual may already be dealing with significant, non-health-related strains in their day-to-day life. Thus, poor health may be a strain that is further intensified when one experiences it in an environment not necessarily conducive to overall well-being.

Notably, the experience of co-occurring health problems is largely associated with an increase in non-serious misconduct, which includes behaviors like possession of an unauthorized object, verbal assault, and being out of place. This suggests that co-occurring health ailments may not provide a strain substantial enough to increase the likelihood of serious misconduct. However, suffering from both a chronic and an acute condition increases the risk of serious misconduct, suggesting that an acute condition may be enough of a strain to increase the risk of serious misconduct even in the face of a chronic condition. In all cases, the greatest increase in misconduct risk is associated with the experience of all three types of conditions together.

We find that combinations of conditions increase the likelihood of misconduct where certain single measures of health condition type do not. After accounting for all forms of conditions and co-occurrence, mental disorder is not associated with serious misconduct alone. This is significant given the body of literature that finds that mental disorder is associated with serious misconduct (e.g., Adams, 1986; Carr et al., 2013; Felson et al., 2012; Houser et al., 2012; Matejkowski, 2017; McCorkle, 1995; Steiner & Wooldredge, 2009; Steiner et al., 2014; Stewart & Wilton, 2014; Wood, 2012; Wood and Buttaro Jr., 2013). However, much of this work does not account for physical conditions and comorbidity. Our results suggest that, compared to healthy individuals in prison, those suffering from a mental disorder that co-occurs with a chronic or acute physical condition are at a significantly higher risk for non-serious misconduct. However, mental disorder appears to only be a risk factor for serious misconduct when it co-occurs with both an acute and chronic condition. Given high rates of physical conditions comorbidity for those with a mental disorder, it may be that the unique strain of a significant physical condition on top of a mental disorder diagnosis is responsible for the increased risk for misconduct, rather than the experience of a mental disorder alone.

The findings also suggest that those dealing with a chronic condition alone are less likely to engage in both types of misconduct. However, when a chronic condition co-occurs with a mental disorder, acute condition, or both, these combinations all increase the risk of non-serious misconduct. Though mental and chronic conditions may not be associated with an increase in particular types of misconduct, co-occurring conditions compound the strain of being unhealthy and may lead to increased risk of misconduct. While previous research has suggested those with a dual diagnosis of a mental disorder and a substance use disorder are more likely to engage in misconduct (Houser et al., 2012; Wood, 2012; Wood and Buttaro Jr., 2013), our findings support expanding the definition of a co-occurring diagnosis to include acute and chronic physical health conditions. This provides a more nuanced understanding of the relationship between health and crime while accounting for physical health processes that researchers may not have considered when only examining the combination of mental disorder and substance abuse.

### Implications for prison healthcare

In addition to improving the lives of those living in prison, our results suggest that efforts to enhance prison healthcare may assist in reducing inmate misconduct. The combination of mental and physical conditions appears to be particularly problematic for misconduct. This cumulative health-related strain may be worsened by poor prison conditions, the inability to access efficient healthcare, and the lack of resources to help cope with being ill. Given this, it may be particularly important for those in prison to receive fast care for acute conditions such as dental problems, illnesses like colds and viruses, and accidental injuries. These health problems likely do not require long-term and specialized care, but appear to be some of the most influential when it comes to misconduct risk. Greater access to outpatient care without long waits and financial burdens for short-term health problems may help to decrease the likelihood of misconduct by removing an important yet amendable strain in the lives of those incarcerated. Relatedly, improvements to medical record management and data retrieval processes across facilities are essential. Improved quality control, accountability, and access to records can ensure that those living in prison, especially if they have spent time in or transferred from another facility, receive appropriate and timely care that is pertinent to their health history.

It is possible that those in prison dealing with acute conditions do not seek out medical services, especially if the services are not readily available or are cost prohibitive. Though these individuals might be in pain or struggling with an illness, they may choose to deal with the symptoms on their own without receiving medical attention. This is problematic if the pain or illness persists and causes continuous discomfort or frustration for the individual. That discomfort may frustrate or anger the person, leading to a “shorter fuse” that may result in certain types of misconduct like a verbal confrontation, insubordination, or citations for not following the rules. Thus, in addition to making outpatient services available for short-term physical health conditions, those in prison should be appropriately educated about all healthcare services available to them so that they are inclined and encouraged to utilize them. All people incarcerated should receive the necessary medical attention and it is possible that failure to do so for acute conditions may create further problems for the individual and the security of the prison.

In addition to an increased focus on improving access to care and utilization for acute conditions, prison healthcare should work to provide improved care for those dealing with co-occurring conditions. These individuals are not only the most ill within the prison population, but they may also be at the highest risk for misconduct if they are dealing with multiple conditions simultaneously. The majority of research related to health and misconduct has focused on mental disorder and its co-occurrence with substance use disorders. Prison healthcare professionals should continue to address this type of comorbidity, while also taking seriously the co-occurrence of mental disorders with physical conditions, even those ailments that officials might deem acute or minor in nature. Although strains are abundant within a prison environment, our results indicate that poor health may be a particularly salient strain when it comes to the day-to-day lives of those in prison and why they engage in misconduct.

Situated within the larger field of epidemiological criminology (Akers & Lanier, 2009), we believe it is increasingly important to assess issues of health and crime alongside one another. Recent research illustrates complex pathways between health behaviors, health outcomes, criminal participation, and exposure to the criminal justice system that require further investigation (Vaughn et al., 2012; Vaughn et al., 2014). Given recent work suggesting that the promotion of health equity may be a pathway towards crime reduction (Jackson & Vaughn, 2018), policymakers and prison healthcare professionals have the opportunity to work towards improvements in inmate healthcare that may serve to also decrease misconduct participation.

### Study limitations and future research

There are certain limitations to this study. We use data from 2004, drawn from a nationally representative sample of state inmates. The results discussed, then, can only be generalized to those individuals in state institutions, rather than federal facilities. Although the data are 14 years old, they represent the most up-to-date data for a nationally representative sample of individuals in state prisons. However, one specific change in prisons has occurred in the last 14 years that has great implications for prison healthcare and potentially the results of this study. In particular, the U.S. prison system has seen the greatest growth in those in prison aged 55 and older, a group more likely to experience poor chronic health than their younger counterparts (Carson, 2016). With a more recent dataset, then, we would expect to see a similar rise in chronic conditions among those in prison. Though smaller, more recent surveys of those in prison have been conducted in the past five years, they are not always nationally representative and do not include in-depth items regarding misconduct and health conditions. Once updated representative data is available that includes sufficient measures of health and misconduct, researchers should examine whether the findings presented here are replicable.

The data are cross-sectional, making it difficult to confirm the direction of causality between inmate health conditions and misconduct. It is possible that causality runs in both directions. It is also possible that misconduct increases the risk of poor health instead of the direction theorized here (see Piquero et al., 2007; Piquero et al., 2011). Despite this possibility, recent research using longitudinal data (Ford, 2014; Kort-Butler, 2017; Stogner & Gibson, 2010, 2011) provides support for a GST argument where health problems lead to greater crime and misconduct. Future studies assessing this relationship among inmates should strive to collect longitudinal data to confirm the directionality of these results. Although longitudinal data collection can be potentially challenged by transfers between facilities, this research can ultimately help determine appropriate policy recommendations to improve health and misconduct within prisons. Similarly, another avenue to better understand the relationship between health conditions and misconduct would be to conduct in-depth interviews with those in prison. These narratives would provide researchers with rich, detailed context surrounding the onset of the health disorder, the potential directionality of the relationship between health and misconduct, as well as how that condition may have influenced his or her misconduct.

Our variables are limited in certain aspects. The misconduct outcomes measured here only account for behaviors that have been reported and sanctioned. Thus, these measures do not capture misconduct that may have taken place but was either not reported or not disciplined.

The data do not include other noted correlates of misconduct such as self-control, association with peers that engage in misconduct, and social control within the prison facility. The measure of acute conditions does not include ailments like joint pain, headaches, migraines, and general pain that are utilized in past studies of acute health and crime (Stogner & Gibson, 2010, 2011). In addition, despite prior research indicating that institutional-level factors influence inmate misconduct (Gendreau et al., 1997; Steiner et al., 2014), our data do not include facility- and institutional-level variables such as population density, facility size, or prison security level. Given that the analyses were restricted to individuals, we could not account for correlated error across respondents nested within the same facility. No details were available regarding where respondents are housed (e.g. specific treatment or medical facilities, solitary confinement), which may influence an individual’s capacity to engage in misconduct. For example, the lack of significant findings between mental disorder alone and serious misconduct might be because those in prison with mental disorders are disproportionately more likely to be housed in solitary confinement (Fellner, 2006; Gilligan & Lee, 2013). Additionally, facility-level characteristics like overcrowding, high security levels and poor confinement conditions may independently contribute to poor health. According to the World Health Organization (2014), overcrowded facilities become breeding grounds for the transmission of communicable, chronic and acute disorders like tuberculosis and influenza. Relatedly, inhumane solitary confinement conditions increase the presence and enhancement of mental disorders (see Metzner & Fellner, 2010) and amplify various physiological symptoms like hypertension, weight loss, lethargy, and insomnia (Haney, 2003; Smith, 2006; Shalev, 2008). Future research should strive to conduct multi-level analyses while taking into account institutional-level factors that may influence misconduct and health separately and impact the relationship between health and misconduct as explored here. Finally, no robust measures of healthcare utilization or medication usage were available in the data, making it difficult to assess how those in prison are being treated for particular conditions.

## Conclusion

The results of this study suggest that co-occurring health conditions are associated with an increase in institutional misconduct. Much of the research on prison health and institutional behavior has focused on the implications of mental disorder diagnosis and substance use disorders. However, the present research contributes to a growing body of evidence suggesting that physical health is also related to misconduct. This has implications for both prison healthcare and future research, suggesting the need to improve accessibility to healthcare in prison, especially for those with acute physical conditions and co-occurring conditions. Encouraging healthcare utilization for short-term health problems and those with co-occurring health conditions may serve to improve overall health in prisons while also reducing misconduct among incarcerated individuals.

## Abbreviations

GST: General Strain Theory
IRR: Incidence rate ratio
MICE: Multiple imputation using chained eqs.
U.S.: United States
VIF: Variance inflation factor

## Footnotes

1 The National Institute on Drug Abuse defines a co-occurring disorder, otherwise known as a dual diagnosis, as “the occurrence of two disorders or illnesses in the same person, either at the same time or with a time difference between the initial occurrence of one and the initial occurrence of the other.”

2 The BJS provides further methodology information on their website at https://www.bjs.gov/index.cfm?ty=dcdetail&iid=275#Methodology.

3 The response rate was 89.1%.

4 Researchers also asked inmates about physical assault of another inmate or staff member, but we did not include assaults due to potential confounding with measures of health problems. It is possible that physical altercations with inmates or staff may result in some of the health conditions measured here and thus they are not included in the final analysis. As a secondary check during initial analysis, we ran a model that included physical assault and found statistically similar relationships between co-occurring conditions and serious misconduct in terms of both coefficient magnitude and statistical significance.

5 About 13% of the analytic sample reports no health problems at all, while 35% have experienced only an acute problem, 4% only a chronic problem, and 3% only a mental problem.

6 General misconduct variance scale (*M* = .77; variance = 1.98).

## Authors information

Daniel C. Semenza is an Assistant Professor in the Department of Sociology, Anthropology, and Criminal Justice at Rutgers University, Camden. His research interests include the connection between health and crime as well as the causes and consequences of violence. His research has been published in *Crime and Delinquency, Criminal Justice and Behavior,* and *Sociological Inquiry.* Email: Daniel.semenza@rutgers.edu

Jessica M. Grosholz is an Assistant Professor of Criminology in the College of Liberal Arts and Social Sciences at the University of South Florida Sarasota-Manatee. Her research interests include prisoner reentry and recidivism, the consequences of mass incarceration, and qualitative research methodologies. Her research has appeared in the *Journal of Criminal Justice*, *Criminal Justice and Behavior*, and *Deviant Behavior*. Email: jgrosholz@sar.usf.edu
